# Clinical Examination and Self-Reported Upper Extremity Impairments in Patients with Long-Standing Type 1 Diabetes Mellitus

**DOI:** 10.1155/2020/4172635

**Published:** 2020-03-11

**Authors:** Kerstin Gutefeldt, Simon Lundstedt, Ingrid S. M. Thyberg, Margareta Bachrach-Lindström, Hans J. Arnqvist, Anna Spångeus

**Affiliations:** ^1^Department of Endocrinology, Linköping University Hospital, Linköping, Sweden; ^2^Department of Health, Medicine and Caring Sciences, Linköping University, Linköping, Sweden; ^3^Department of Rheumatology and Department of Biomedical and Clinical Sciences, Linköping University, Linköping, Sweden; ^4^Division of Nursing Sciences, Department of Health, Medicine and Caring Sciences, Linköping University, Linköping, Sweden; ^5^Department of Biomedical and Clinical Sciences, Linköping University, Linköping, Sweden; ^6^Department of Acute Internal Medicine and Geriatrics, Linköping University Hospital, Linköping, Sweden

## Abstract

**Aim:**

The aims of the current study were (1) to determine the prevalence of upper extremity impairments (UEIs) in patients with type 1 diabetes by clinical investigation; (2) to investigate if self-reported impairments were concordant with clinical findings and if key questions could be identified; and (3) to investigate if answers to our self-reported questionnaire regarding UEIs are reliable.

**Methods:**

Patients with type 1 diabetes were invited to participate in a cross-sectional study of clinical and self-reported (12 items) UEIs in adjunction to ordinary scheduled clinical visit. Before the visit, a questionnaire on UEIs was filled in twice (test-retest) followed by clinical testing at the planned visit.

**Results:**

In total, 69 patients aged 45 ± 14 years and with diabetes duration 26 ± 15 were included in the study. In the clinical examination, two-thirds (65%) of the patients showed one or more UEI, with failure to perform hand against back as the most common clinical finding (40%) followed by positive Phalen's test (27%), Tinel's test (26%), and Prayer's sign (24%). UEIs observed by clinical examination were often bilateral, and multiple impairments often coexisted. Self-reported shoulder stiffness was associated with impaired shoulder mobility and with Prayer's sign. Self-reported reduced hand strength was associated to lower grip force, Prayer's sign, trigger finger, fibrosis string structures, and reduced thenar strength as well as reduced shoulder mobility. In addition, self-reporting previous surgery of carpal tunnel and trigger finger was associated with several clinical UEIs including shoulder, hand, and finger. The test-retest of the questionnaire showed a high agreement of 80-98% for reported shoulder, hand, and finger impairments.

**Conclusion:**

UEIs are common in type 1 diabetes. Self-reported shoulder stiffness and reduced hand strength might be used to capture patients with UEIs in need of clinical investigation and enhanced preventive and therapeutic strategies, as well as rehabilitative interventions.

## 1. Introduction

Upper extremity impairments (UEIs) which mainly involve the connective tissue of the shoulder, hand, and fingers are common in patients with type 1 diabetes (T1D) [1–4].

Frozen shoulder, limited joint mobility, carpal tunnel syndrome, trigger finger, and Dupuytren's contracture are all conditions more prevalently observed in diabetes [5–8]. Diabetes also affects muscle tissue, and impaired muscle function has been observed in diabetes [9–11]. Pathogenesis is assumed multifactorial with risk factors such as increasing age, female gender, longer diabetes duration, higher BMI, poor glycemic control, and presence of micro- and macrovascular complications [6]. An attractive hypothesis with some support is that increased advanced glycation end products in collagen (AGEs) cause the UEI in diabetes [6, 12, 13]. UEIs are associated with physical disability and impaired health-related quality of life (HRQOL) [1, 6, 14–16].

In a previous publication, we used a questionnaire to explore prevalence of UEIs in all patients with long duration of T1D living in the Southeast region of Sweden and found that UEIs are very common [17]. However, self-reported impairments might differ from clinical findings. Furthermore, UEIs can cause physical and mental disability and are associated with impaired work ability urging for more awareness and rehabilitating interventions [15, 16, 18, 19]. UEIs are related to other diabetes complications such as neuropathy and cardiovascular disease and to mortality and thus add information to the overall risk profile [6, 20]. UEIs are underestimated, and there is a need to develop clinically useful key questions to capture UEIs in diabetes at annual check-up in today's diabetes care.

The aims of the current study were (1) to determine the prevalence of upper extremity impairments (UEIs) in patients with type 1 diabetes by clinical investigation; 2) to investigate if self-reported impairments were concordant with physical findings and if key questions could be identified; and (3) to investigate if our self-reported questionnaire regarding UEIs is reliable.

## 2. Material and Methods

### 2.1. Design and Inclusion

This cross-sectional study was conducted in Linköping, Sweden, between 2017 and 2018. Patients with type 1 diabetes (T1D) aged 18-69 years who had a scheduled appointment at the outpatient diabetes clinic at Linköping University Hospital were invited by postal mail to participate. The invitation included a questionnaire on upper extremity impairments. In case of approval, patients were asked to send the questionnaire and a signed consent to the clinic. Furthermore, patients were asked to fill in the questionnaire a second time prior to the clinical examination at the hospital. The mean time span between the first and the second questionnaire was 13 ± 8 days. In total, 69 patients participated in the study out of 200 invited.

### 2.2. Self-Reported Questionnaire

The self-reported questionnaire about UEIs has been published previously [17]. It included questions about shoulder pain and stiffness and specific questions regarding impairments in arms, hands, and fingers (Table 1). There were also questions about previous surgery for trigger finger and carpal tunnel syndrome, reduced hand strength, and previous fractures. Answers to the first questiosnnaire were used to compare the result of the questionnaire with that of the clinical examination, regarding all our clinical assessments of shoulder, hands, and fingers. Data from the first and second questionnaires was used to analyze test-retest, i.e., the reproducibility of the answers.

### 2.3. Clinical Examination

**Figure 1:**
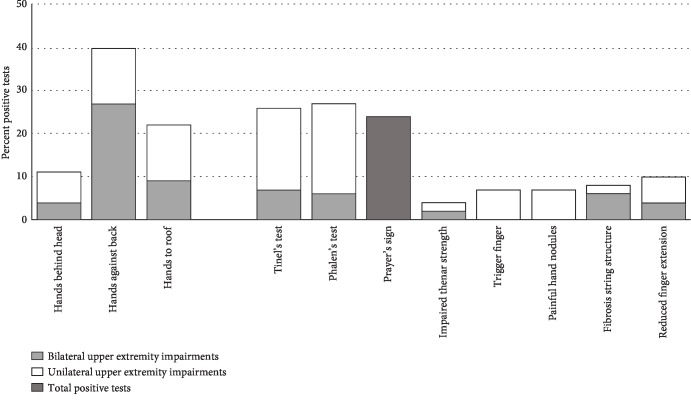
Prevalence of clinical examined uni- and bilateral upper extremity impairments.

A study specific protocol, related to the questionnaire, was designed for the clinical examination. Guidelines for the examination and definitions for clinical findings were included in the protocol. All examinations were tested on both sides (left and right) in all 69 patients. In the tests, the clinical finding was considered positive or negative, e.g., in Phalen's test, the results were reported as either (1) uni- or bilateral (Figure 1) or (2) present (including both uni- and bilateral) or not (i.e., neither in the left nor in the right side). The latter was used when correlating clinical results to each other and to self-reported impairments (questionnaire).

Shoulder mobility was tested regarding active range of motion for each arm with three functionally important movement tests performed in a standing position (e.g., “hands behind head” performed by keeping the elbow in the frontal plane (positive test if unable to place the hand behind the head), “hands against back” (positive if unable to reach lower level of scapula), and “hands to roof” (positive if unable to abduct the shoulder). These three tests were performed once. Furthermore, active shoulder mobility was measured in supine position with a plastic goniometer for flexion, extension, abduction, inward, and outward rotation. The tests were performed twice on each side, and a mean value was calculated. The lowest mean value achieved, i.e., from left or right, was used in the analysis.

Phalen's and Tinel's tests were applied to check for sensation of tingling or numbness in hands and fingers. Phalen's test was performed by flexing both wrists and elbows in a 90-degree position. Tinel's test consisted of tapping lightly, using the index finger over the median nerve (proximal palmar area and over the wrist) with the patients' wrist in extension. If the test was associated with paresthesia in the entire finger area innervated by the median nerve after 30-60 seconds, it was considered positive.

Thenar strength was tested by thumb abduction. Inability to abduct the thumb against the investigator applied resistance was defined as impaired thenar strength.

Trigger finger was investigated by asking the participants to tighten their fists around the examiner's finger placed over the metacarpophalangeal joints. If any finger snapped or got stuck in a flexed position during the movement and was associated to pain, the outcome was considered positive.

Reduced finger extension was examined visually and by palpation in two assessments: (a) presence of nodules or fibrosis string structures in the palmar side of digit 4 or 5 and (b) inspection if those fingers were notedly flexed and unable to extend.

Painful hand nodules were examined by palpation and were considered positive if associated to pain.

Prayer's sign was tested by placing palmar surfaces flat to one another with extended wrists and with 90-degree flexed elbows. The test was considered positive if the patient was unable to fully close gaps between the opposed palms and fingers when pressing them together.

Previous carpal tunnel or trigger finger surgery was confirmed by visual inspection regarding scars in palms and on fingers. In addition, findings were discussed with the patients.

Grip force was evaluated using the device Grippit (Nr: D9110G, AB Detektor, Göteborg, Sweden). Reference values were attained from a previous Norwegian study [21]. The analysis was performed separately for female and male participants. The mean value for both left and right hands was recorded. Thirteen percent of the patients were left-handed. There was no significant difference in grip force between left- and right-handed individuals. The lowest value achieved in either left or right hand was used in the analysis.

Two examiners performed the clinical investigation. Prior to the examinations, they were trained by an occupational therapist and a physiotherapist specialized in upper extremity examination techniques.

### 2.4. Risk Factors

Data on the presence of retinopathy (defined as laser-treated proliferative retinopathy), cardiovascular disease (previous stroke, myocardial infarction/angina, or present peripheral arterial disease), and nephropathy (micro- or macroalbuminuria) was retrieved from medical records. Peripheral neuropathy was investigated with 2 tests performed once on both right and left feet: (1) monofilament (10 g) was tested at the plantar surface of the foot (at the hallux and at the metatarsophalangeal joint digits 1 and 5). The test was considered positive if there is loss of sensation in ≥1 location; (2) a 128 Hz tuning fork was used to examine vibration perception at the dorsum of the interphalangeal joint of the hallux and at the medial malleolus. The test was considered positive if there is loss of sensation in ≥1 location.

### 2.5. Laboratory Methods

HbA1c was analyzed using a TOSOH G7 automated haemoglobin analyzer (Tosoh Bioscience, Tokyo, Japan).

### 2.6. Statistics

Demographic and clinical characteristics of patients with or without upper extremity impairments were compared using independent *T*-test for numerical and Chi-square test for categorical variables, presented as mean and as percentage, respectively. To evaluate test-retest reliability of self-reported impairments, categorical variables were analyzed with descriptive statistics (frequency) to receive a concrete percentual agreement of items in the questionnaire. If a patient's first answer to a question was equal to the second time answering the same question, it was defined as an agreement. To determine relations between self-reported symptoms and clinical findings of UEIs, we used Pearson's correlation analysis. Grip force assessed by Grippit (Newton) was analyzed separately for males and females because of the established gender differences [21]. All tests were performed using “IBM SPSS Statistics,” version 25.0 for windows, released in 2017, Armonk, NY by IBM Corp. In all tests, the statistical significance level was set to a *p* value of <0.05.

### 2.7. Ethics

The ethical review board of Linköping University hospital approved of the study on the 15^th^ of February 2017, Dnr:2017/72-32. All the patients assigned a term of consent before the enrolment.

## 3. Results

### 3.1. Participants' Characteristics

The study group consisted of 69 patients, 51% females. The mean age of the participants was 45 ± 14 years, and the mean diabetes duration was 26 ± 15 (median 25) years, with 81% of patients having ≥10 years diabetes duration. BMI was 27 ± 5 kg/m^2^, and mean HbA_1c_ was 59 ± 12 mmol/mol (IFCC), 7.5 ± 1.1% (NGSP). As regards risk factors, 29% had neuropathy, 17% laser-treated retinopathy, 9% nephropathy, and 6% cardiovascular disease.

### 3.2. Prevalence of Self-Reported Upper Extremity Impairments

We found that the most common self-reported impairment was shoulder stiffness (49%), followed by shoulder pain (44%) and hand paresthesia (40%). As shown in Table 1, the self-reported impairments were to a high extent bilateral. Self-reported shoulder pain and stiffness correlated positively to each other *r* = 0.612, *p* value < 0.001. In addition, reported shoulder stiffness positively correlated to hand stiffness (0.254, *p* = 0.039), and reported hand stiffness correlated positively to hand pain *r* = 0.554, *p* value < 0.001.

Test-retest analysis of the questionnaire showed a percentual agreement between 80.3 and 98.4% for self-reported shoulder, hand, and finger impairments.

### 3.3. Prevalence of Upper Extremity Impairments by Clinical Examination

In total, two-thirds (65%) of the patients had one or more uni- or bilateral clinical signs of UEIs (i.e., unable to perform “hand against back,” “hand behind head,” or “hand to roof”; positive Phalen's, Tinel's, or Prayer's sign; painful hand nodules; reduced thenar strength, trigger finger; and/or reduced finger extension). The most common finding was a positive shoulder examination test “hands against back,” which was found positive in 40% of the patients (Figure 1). One out of four patients had a positive Tinel or Phalen test. Almost as common was Prayer's sign, which was positive in 24% of the patients. As shown in Figure 1, most clinical findings were commonly bilateral except for trigger finger and painful hand nodules. Thirteen percent of patients had scars from previous carpal tunnel surgery, and of those, 56% had previous bilateral surgery. Similarly, 13% of patients had scars from previous trigger finger surgery (56% bilaterally).

Shoulder mobility measurements were similar in males and females except for shoulder abduction and shoulder outward rotation where males had greater abduction 163° ± 26° vs 146° ± 41° (*p* value 0.038) and outward rotation 70° ± 17° vs 59° ± 30 (*p* value 0.055), respectively. The mean grip force for all participants was 231 ± 122 Newton. Males were stronger than females: 300 ± 123 and 165 ± 76, respectively (*p* value < 0.001).

Coexisting impairments in shoulder were common, and in all, but 2 patients, it included “hands against back.” Of those patients that had any of the three shoulder impairments, “hand against back,” “hand behind head,” or “hand to roof” (43%), 22% were positive for one, 12% for two, and 9% for all three tests. Of those patients with either Tinel's and or Phalen's test positive (36%), 17% were positive for both tests.

#### 3.3.1. Correlation between Upper Extremity Impairments Based on Clinical Examination

The clinically evaluated impairments of shoulder mobility were all related to each other. “Hands against back” was associated with “hands to roof” *r* = 0.553, *p* value < 0.01 and to “hands behind head” *r* = 0.881, *p* value < 0.01. Furthermore, shoulder examination, e.g., “hands against back” associated with scars from previous trigger finger (*r* = 390, *p* value < 0.01) and carpal tunnel surgery (*r* = 0.390, *p* value < 0.05). All 3 shoulder exercises were negatively correlated to mobility (i.e., flexion, extension, abduction, outward and inward rotation).

Prayer's sign was also associated to several shoulder, hand, and finger impairments, e.g., “hands against back” (*r* = 0.485, *p* value < 0.01), grip force (*r* = −0.267, *p* value < 0.05), and trigger finger (*r* = 0.241, *p* value < 0.05).

### 3.4. Relation between Clinical Examination versus Self-Reported Upper Extremity Impairments

As shown in Table 2, patients who self-reported shoulder stiffness had significantly reduced mobility (flexion, extension, abduction, inward, and outward rotation) than those who reported no stiffness. Greatest discrepancy was observed for abduction 136° ± 32° vs 171° ± 32° and outward rotation 53° ± 24 vs 76° ± 20°, respectively, *p* value < 0.001. When analyzing relation between clinical versus self-reported impairments, reported shoulder stiffness showed significant association to inability to perform the three clinical tests “hands behind head” (*r* = 0.429, *p* value < 0.01), “hands against back” (*r* = 0.546, *p* value < 0.01), and “hands to roof” (*r* = 0.501, *p* value < 0.01), as well as to worse shoulder mobility in all five axes (Table 3). A similar result was observed for self-reported shoulder pain (Table 3). Additionally, self-reported shoulder stiffness was associated to a positive Prayer's sign (*r* = 0.354, *p* value < 0.01) (Table 3).

Grip force was significantly lower in both female and male patients who reported reduced hand strength compared to those who reported normal hand strength (males 181 ± 92 vs 336 ± 108 Newton, *p* value 0.001 and females 124 ± 51 vs 190 ± 79 Newton, *p* value 0.014 (Table 2)). Self-reported “reduced hand strength” was associated with impaired grip force, positive Prayer's sign, reduced thenar strength, trigger finger, and present fibrosis string structures (Table 4). Furthermore, self-reported “reduced hand strength” was associated with reduced shoulder mobility (Table 3).

Patients reporting previous trigger finger surgery more often showed clinical findings such as Prayer's sign, impaired thenar strength, trigger finger, and present painful nodules (Table 4). Furthermore, these patients showed reduced shoulder mobility, including shoulder extension and inward and outward rotation as well as “hands behind head” and “hands against back” (Table 3).

## 4. Discussion

Up to date, UEIs in type 1 diabetes have received little attention in routine patient clinic visits and there is no standardized procedure to capture patients with UEIs. In this cross-sectional study, we tested the ability of a structured questionnaire to recognize upper extremity impairments in patients with type 1 diabetes in comparison to clinical examination. Examined by a test-retest procedure, the answers to the questionnaire were highly reproducible. In our study group, with mainly patients with long diabetes duration (81% ≥10 years), UEIs were very common with a prevalence over 60% both as reported in the questionnaire and as observed in the clinical examination. Symptoms of shoulder impairment (stiffness and pain) were most frequently reported and closely associated with clinical impaired shoulder function. Also, self-reported hand and finger impairments were associated with impaired clinical function. These data suggest that a self-reported questionnaire can be a valuable tool to screen for UEIs.

In the clinical examination of the shoulder, 40% of the patients showed impaired mobility, “hands against back,” which is compatible but not specific for frozen shoulder as well as the observed reduced mobility in several planes [4, 22]. Previous reports regarding prevalence of frozen shoulder use somewhat different diagnose criteria [6, 12, 23] but involve pain, stiffness, and impaired shoulder range of motion. Numerous shoulder examination techniques have been evaluated in order to diagnose shoulder conditions; however, none of the tests is considered a 100% sensitive or specific [24–29]. Larkin et al. defined frozen shoulder by self-report and shoulder flexion measurement and reported a prevalence of 31% of the patients [6]. A recent Sardinian study reported adhesive capsulitis in 35% of the patients [12]. Our findings highlight shoulder impairment in diabetes and stress the importance to find patients with shoulder immobility at an early stage to possibly prevent further deterioration of pain and stiffness [30].

Signs of carpal tunnel syndrome, tested with Tinel's and Phalen's tests, were positive in 26% and 27% of the patients, respectively, thus concordant with Larkin et al. who reported a prevalence of 30%. Self-reported tingling fingers or waking up to tingling fingers, suggestive of carpal tunnel syndrome, was associated with each other but not associated with either a positive Tinel's or Phalen's test. A possible explanation is that these self-reported symptoms might also represent diabetic neuropathy (DN) and not only carpal tunnel syndrome and thus will not be positive in physical examination by Tinel and Phalen tests. It should be pointed out that these questions had somewhat lower reproducibility than other questions in the test-retest evaluation.

A positive Prayer sign was found in 24% of the patients comparative to a prevalence of 22% reported by Larkin et al. [6]. Prayer's sign, indicative of LJM, was associated with many other clinically evaluated shoulder hand and finger impairments, implicating that patients with diabetes commonly display a general stiffness in the upper extremities [23].

### 4.1. Self-Reported Impairment vs Clinical Exam

We have shown that self-reported shoulder stiffness and pain significantly correlate to impaired clinically evaluated shoulder mobility using three simple shoulder exercises and goniometer measurements. The aim of this study was to seek easily applicable tools, i.e., key questions, to capture UEIs on a regular diabetes checkup. In this context, our questions on shoulder pain or stiffness showed correlation with mobility impairment in all examined shoulder tests. Thus, asking patients about shoulder pain and stiffness might help clinicians to find patients with shoulder impairments in need of further evaluation.

Grip force is known to be lower in women compared to men [21]. We found that regardless of gender, patients self-reporting reduced hand strength had significantly lower objective grip force. Furthermore, they associated with the presence of several clinical hand and finger impairments, i.e., Prayer's sign, trigger finger, fibrosis string structures, and reduced thenar muscle strength as well as reduced shoulder mobility. Following this finding, a question of whether the patient experiences reduced hand strength might help the clinician to locate risk patients of clinical UEIs.

Self-reported previous surgery for trigger finger and carpal tunnel syndrome was associated with several present clinical UEIs, in shoulder, hand, and finger, indicating an increased risk profile in general for upper extremity impairments of these patients. This is also strengthened by the fact that impairments often are bilateral, and multiple impairments often coexist. Taken together, this indicate that these patients, with previous surgery for trigger finger or carpal tunnel syndrome, constitute a high risk for other UEIs and might need extra clinical focus.

### 4.2. Key Questions to Capture UEIs

We sought to find key questions to identify patients with UEIs. The usefulness of current self-reported questionnaire to capture UEIs proved sensitive to identify shoulder and certain hand impairments. The questions “*Do you experience shoulder stiffness*,” “*Do you experience shoulder pain*,” and “*Do you experience reduced hand strength*” could be useful to find patients who need further evaluation. Furthermore, previous surgery of trigger finger or carpal tunnel syndrome could be valuable to record, either self-report or from case record.

### 4.3. Limitations

There are some limitations to consider. Our study included type 1 diabetes patients consecutively recruited at the outpatient clinic at the time of the study. No control group was included in the study. The acceptance rate of 35% is rather low, and there is a risk of selection bias, where patients having impairment might be more motivated to participate. Clinical test only included basic tests available at the outpatient clinic. To make a correct diagnosis of carpal tunnel syndrome, other assessments such as neurophysiology facilitate diagnostics [31].

## 5. Conclusions

UEIs are common and underestimate complications in type 1 diabetes with long duration. Presence of UEIs should be assessed parallel to other complications at annual diabetes checkups. Key questions regarding shoulder stiffness and reduced hand strength in addition to previous surgery for trigger finger or carpal tunnel syndrome may be helpful to capture patients with UEIs in need of clinical investigation and enhanced preventive and therapeutic strategies as well as rehabilitative interventions.

## Figures and Tables

**Table 1:** Prevalence of self-reported upper extremity impairments.

Questionnaire	Reported impairments		
	Total number of patients with impairment (%)	Bilateral impairments (%)	Unilateral impairments (%)
Do you have pain/ache in shoulder joints? (shoulder pain)	30 (44)	19 (28)	11 (16)
Do you have stiffness in shoulder joints? (shoulder stiffness)	33 (49)	26 (38)	7 (10)
Do you have pain/ache in the hand or forearm? (hand pain)	24 (35)	18 (27)	6 (9)
Are your hand or forearm stiff? (hand stiffness)	16 (24)	12 (18)	4 (6)
Do you have tingling or loss of sensation/numbness in fingers? (hand paresthesia)	27 (40)	19 (28)	8 (12)
Do you wake up at night due to pain or tingling/loss of sensation in hands? (wake up to tingling fingers)	18 (26)	13 (19)	5 (7)
Do you experience weakness in the hand? (reduced hand strength)	20 (29)	14 (21)	6 (9)
Have you ever had surgery for carpal tunnel syndrome (nerve entrapment in wrist)? (previous CT surgery)	10 (14)	6 (9)	4 (6)
Does it ever happen that one of the fingers “lock” when trying to bend it? (finger locking)	15 (22)	7 (10)	8 (12)
Do you have “tendon nodules” in the palm? (hand nodules)	10 (15)	5 (7)	5 (7)
Have you ever had surgery for “tendon nodules” or a stricture in the tendon sheath of the palm? (previous TF surgery)	6 (9)	4 (6)	2(3)
Have you had trouble straightening any finger/fingers? (flexed finger)	6 (9)	5 (7)	1 (2)

**Table 2:** Clinical examined shoulder mobility and grip force in patients with and without self-reported shoulder stiffness and reduced hand strength.

	All participants (*n* = 69)	Patients self-reporting		*p* value
Shoulder mobility (goniometer)^¤^		Having shoulder stiffness (*n* = 33)	No shoulder stiffness (*n* = 35)	
Flexion shoulder (degrees)	162 ± 16	153 ± 17	170 ± 8	*<0.001*
Extension shoulder (degrees)	60 ± 9	56 ± 10	63 ± 6	*0.001*
Abduction shoulder (degrees)	154 ± 35	136 ± 32	171 ± 32	*<0.001*
Inward rotation shoulder (degrees)	43 ± 17	36 ± 15	51 ± 16	*<0.001*
Outward rotation shoulder (degrees)	64 ± 25	53 ± 24	76 ± 20	*<0.001*
Grip force (Grippit)^		Having reduced hand strength (*n* = 20)	No reduced hand strength (*n* = 48)	
All (Newton)	231 ± 122	147 ± 74	269 ± 120	*<0.001*
Male (Newton)^#^	300 ± 123	181 ± 92	336 ± 108	*0.001*
Female (Newton)^#^	165 ± 76	124 ± 51	190 ± 79	*0.014*

Data are presented in mean ± SD. *p* value analyzed between patients with and without shoulder stiffness and reduced hand strength, respecitvely. ^¤^The lowest obtained value in degrees from the five goniometer measurement axes left or right shoulder; flexion, extension, abduction, and inward and outward rotation, respectively. ^The lowest obtained Grippit value (Newton) for either left or right hand is presented. ^#^Male *n* = 34 (8 reported reduced hand strength and 26 not), female *n* = 35 (12 reported reduced hand strength, 22 not).

**Table 3:** Correlation between clinical examination of the shoulder and self-reported upper extremity impairments.

		Clinical examination SHOULDER							
		Hands behind head	Hands against back	Hands to roof	Flexion	Extension	Abduction	Inward rotation	Outward rotation
Questionnaire	Shoulder pain	*0.320* ^∗∗^	*0.449* ^∗∗^	*0.409* ^∗∗^	*-0.477* ^∗∗^	*-0.247* ^∗^	*-0.364* ^∗∗^	*-0.385* ^∗∗^	*-0.430* ^∗∗^
	Shoulder stiffness	*0.429* ^∗∗^	*0.546* ^∗∗^	*0.501* ^∗∗^	*-0.562* ^∗∗^	*-0.398* ^∗∗^	*-0.492* ^∗∗^	*-0.442* ^∗∗^	*-0.456* ^∗∗^
	Hand pain	ns	ns	ns	ns	ns	ns	ns	ns
	Hand stiffness	ns	ns	ns	*-0.260* ^∗^	ns	ns	ns	ns
	Tingling fingers	ns	ns	ns	ns	ns	ns	ns	ns
	Wake up to tingling fingers	ns	ns	ns	ns	ns	ns	ns	ns
	Reduced hand strength	ns	*0.284* ^∗^	*0.340* ^∗∗^	*-0.267* ^∗^	ns	ns	ns	*-0.372* ^∗∗^
	Finger locked	ns	ns	ns	ns	ns	ns	ns	*-0.243* ^∗^
	Hand nodules	ns	ns	ns	ns	ns	ns	*-0.312* ^∗^	*ns*
	Flexed finger	ns	ns	ns	ns	ns	ns	ns	ns
	Previous CT surgery	ns	*0.342* ^∗∗^	*0.302* ^∗^	ns	ns	ns	ns	*-0.296* ^∗^
	Previous TF surgery	*0.335* ^∗∗^	*0.392* ^∗∗^	ns	ns	*-0.328* ^∗∗^	ns	*-0.257* ^∗^	*-0.250* ^∗^

Data is presented as correlation coefficient “*r*”, 1 = present and 0 = absent. Significance levels are ^∗^*p* < 0.05 and ^∗∗^*p* < 0.01.

**Table 4:** Correlation between clinical examination of the hand/fingers and self-reported upper extremity impairments.

		Clinical examination HAND/FINGER								
		Grip force (Newton)	Tinel's test	Phalen's test	Prayer's sign	Thenar strength	Trigger finger	Painful hand nodules	Fibrosis string structure	Reduced finger extension
Questionnaire	Shoulder pain	*-0.238* ^∗^	ns	ns	ns	ns	ns	ns	ns	ns
	Shoulder stiffness	ns	ns	*0.273* ^∗^	*0.354* ^∗∗^	ns	ns	ns	ns	ns
	Hand pain	*-0.325* ^∗∗^	ns	ns	*0.307* ^∗^	ns	*0.380* ^∗∗^	ns	ns	ns
	Hand stiffness	*-0.352* ^∗∗^	*0.292* ^∗^	ns	ns	*0.313* ^∗∗^	*0.374* ^∗∗^	ns	ns	ns
	Tingling fingers	ns	ns	ns	*0.248* ^∗^	ns	ns	ns	ns	ns
	Wake up to tingling fingers	ns	ns	ns	ns	ns	ns	ns	ns	ns
	Reduced hand strength	*-0.461* ^∗∗^	ns	ns	*0.350* ^∗∗^	*0.269* ^∗^	*0.435* ^∗∗^	ns	*0.311* ^∗^	ns
	Finger locked	ns	*0.263* ^∗^	ns	*0.284* ^∗^	ns	*0.318* ^∗∗^	ns	*0.256* ^∗^	ns
	Hand nodules	ns	ns	ns	ns	ns	ns	ns	ns	ns
	Flexed finger	*-0.285* ^∗^	ns	ns	*0.369* ^∗∗^	*0.250* ^∗^	*0.506* ^∗∗^	*0.306* ^∗^	ns	ns
	Previous CT surgery	ns	ns	ns	*0.257* ^∗^	ns	ns	ns	ns	ns
	Previous TF surgery	ns	ns	ns	0.456^∗∗^	*0.252* ^∗^	*0.308* ^∗^	*0.507* ^∗∗^	ns	ns

Data is presented as correlation coefficient “*r*”, 1 = present and 0 = absent. Significance levels are ^∗^*p* < 0.05 and ^∗∗^*p* < 0.01.

## Data Availability

The SPSS data used to support the findings of this study are available from the corresponding author upon request.
